# Refractory Arrhythmias as a Potential Indicator of Underlying Cardiac Amyloidosis: A Case Report

**DOI:** 10.7759/cureus.41760

**Published:** 2023-07-12

**Authors:** Matthew G Colas, Christelle R Azolin, Juan Gabriel Jimenez, Muhammad A Aziz

**Affiliations:** 1 Internal Medicine, Florida International University, Herbert Wertheim College of Medicine, Miami, USA

**Keywords:** refractory arrhythmias, autonomic dysregulation, restrictive cardiomyopathy, amyloidosis (al), cardiac amyloidosis, atrial fibrillation

## Abstract

Primary (AL) amyloidosis is a rare multisystemic disorder that occurs approximately in 9.7-14.0 cases per million per year in the United States. A late diagnosis of amyloidosis can decrease the chance of survival to less than three years. With the intention to diagnose future cases of AL amyloidosis early in clinical presentation, we describe a case of a 64-year-old female who had presented to the hospital for a pre-liver transplant workup for presumed end-stage liver disease secondary to nonalcoholic steatohepatitis (NASH). Pre-transplant electrocardiogram (ECG) findings were significant for atrial fibrillation that was unable to resolve with synchronized cardioversion. Two previous cardioversions attempted in the preceding three years with amiodarone proved unsuccessful. Following her ECG, an endoscopy and colonoscopy were completed that demonstrated a lesion within the gastric mucosa along with two polyps in the transverse colon and ascending colon. Pathology for these lesions revealed amyloidosis in all biopsy sites, which was followed by a bone marrow biopsy also confirming AL amyloidosis and proliferative monoclonal B lymphocytes. A cardiac magnetic resonance imaging (MRI) proceeded to gather more information on the systemic extent of the patient’s amyloidosis, which showed signs consistent with cardiac infiltration of amyloid. The patient was discharged with at-home hospice care and later decided to pursue chemotherapy, ultimately expiring from end organ failure. We conclude that failed cardioversion in a patient with persistent atrial fibrillation can be a clinical and diagnostic marker in suspecting a diagnosis of amyloidosis. Thus, we encourage clinicians to consider systemic amyloidosis in the assessment of unsuccessful cardioversion in these patient presentations for the initiation of treatment early on in the disease course.

## Introduction

Amyloidosis is classified as a multisystemic disorder that is capable of affecting the functionality of various organs such as the liver, kidneys, intestines, heart, etc. [1]. It is considered a rare diagnosis that is found in 9.7-14.0 cases per million per year [2]. The most common type of amyloidosis, AL amyloidosis, is the most severe and deadly. AL amyloidosis can arise from monoclonal B cell dyscrasia and involves the immunoglobulin light or heavy chain as the insoluble protein subunit that accumulates extracellularly, leading to impaired organ function and cellular death [1,3,4]. The clinical presentation of AL amyloidosis generally includes nonspecific symptoms such as fatigue, unintentional weight loss, and orthostatic hypotension [1]. Some more specific signs include macroglossia or periorbital purpura, however, these findings are only present in 15% of patients that have AL amyloidosis [1]. Other systemic signs of AL amyloidosis include diastolic dysfunction due to restrictive cardiomyopathy, atrial fibrillation, hepatomegaly, carpal tunnel syndrome, and autonomic dysfunction [1]. However, the majority of the patients affected by AL amyloidosis expire from heart failure or uremia [4]. On average, the prognosis is very poor in AL amyloidosis [4]. The average life expectancy is less than three years in patients diagnosed with AL amyloidosis with less than 5% of individuals surviving past 10 years [4]. Certain therapies have demonstrated an overall median survival longer than 10 years [4]. Once amyloidosis invades the myocardium, the prognosis drops to less than one year [5]. Delayed diagnosis likely contributes to poor prognosis among patients, despite the increased number of treatment options [5]. In this study, we describe a case of a patient who presented with systemic amyloidosis to illustrate the potential of further clinical workup in persistent atrial fibrillation with failed cardioversion.

## Case presentation

**Figure 1:**
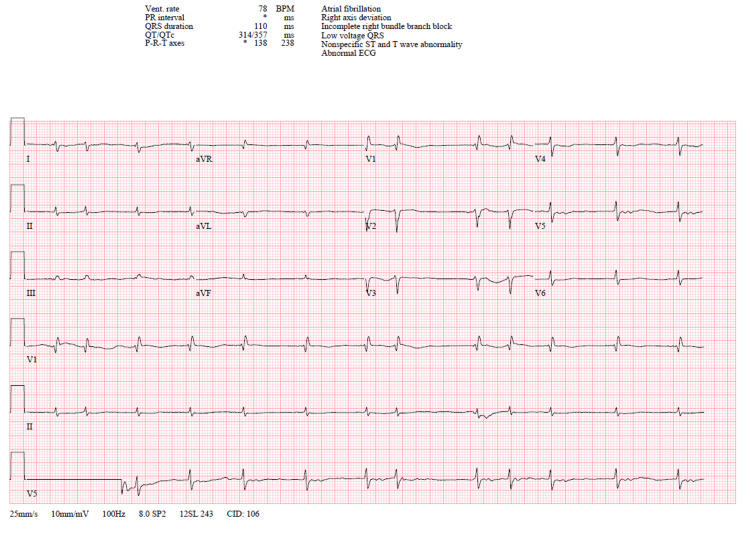
Electrocardiogram This is a 12-lead electrocardiogram (ECG) with atrial fibrillation, right axis deviation, incomplete right bundle branch block, and possible right ventricular hypertrophy.

In February of 2023, a 64-year-old female with a history of hypertension, hyperlipidemia, heart failure, and presumed nonalcoholic steatohepatitis (NASH) presented to the hospital for a liver transplant workup due to end-stage liver disease from presumed NASH. Electrocardiogram (ECG) on current admission was significant for atrial fibrillation with evidence of low voltage, nonspecific T-wave changes, and incomplete right bundle branch blocks (Figure 1). The patient had unresolved atrial fibrillation since 2019, where she underwent two failed cardioversions with amiodarone. Rate control with beta-blockers proved unsuccessful. She denied any previous tobacco or alcohol use. The course of her disease started as arrhythmias that progressed to liver involvement and then significant weight loss. Physical examination was positive for irregular rate and rhythm, peripheral edema, ascites, orthostatic hypotension, asterixis in the right hand greater than the left, and periorbital purpura.

**Figure 2:**
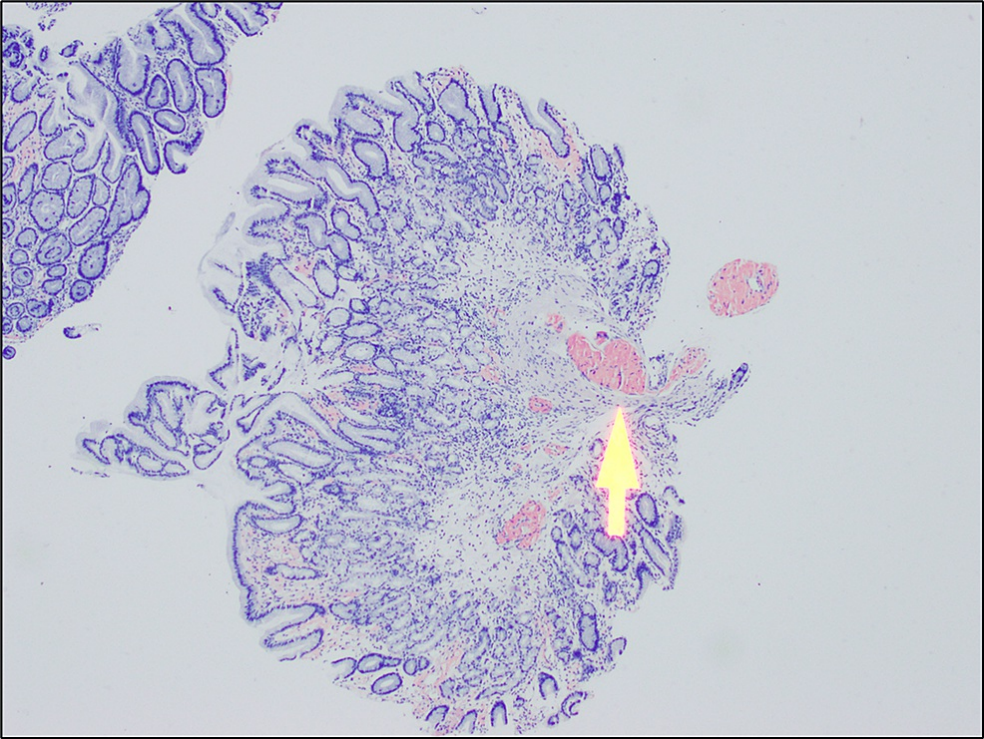
Gastrointestinal Biopsy Congo-red stain is positive for amyloid deposits (shown in arrow).

During the patient's most recent hospitalization, she underwent gastrointestinal and cardiological evaluation as part of the pre-transplant evaluation for presumed NASH. A gastrointestinal assessment was completed prior to cardioversion to assess for potential esophageal varices. On endoscopy evaluation, a nonbleeding cratered gastric ulcer was discovered, along with two polyps in the transverse colon and ascending colon. The lesions were excised and sent for histopathological examination. Following endoscopy, the patient underwent synchronized cardioversion at 200J twice but immediately returned to atrial fibrillation. Amiodarone was excluded due to the patient’s history of cirrhosis. Apixaban 5 mg twice a day was continued for stroke prevention. Pathology report of the gastric mucosa and intestinal tract returned positive for congo red staining, indicative of amyloidosis (Figure 2). This finding prompted further investigation into amyloidosis to understand the extent of disease involvement for our patient.

**Figure 3:**
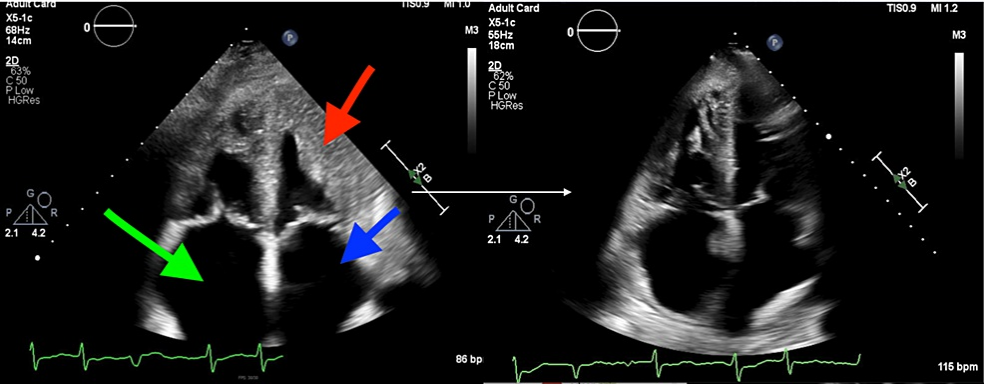
Transthoracic Echocardiogram A transthoracic echocardiogram showed concentric remodeling of the left ventricle (red arrow). The left atrium is mildly dilated (red arrow) and the right atrium (green arrow) is severely dilated.

**Figure 4:**
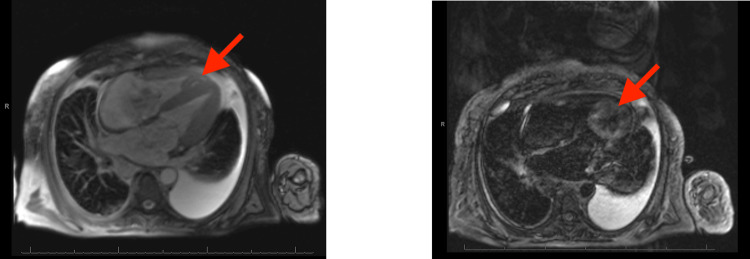
Cardiac MRI T2 weighted images in a short axis plane, resting images were completed with intravenous (IV) multihance contrast. A few focal areas of enhancement were located within the septum (shown in arrow).

**Figure 5:**
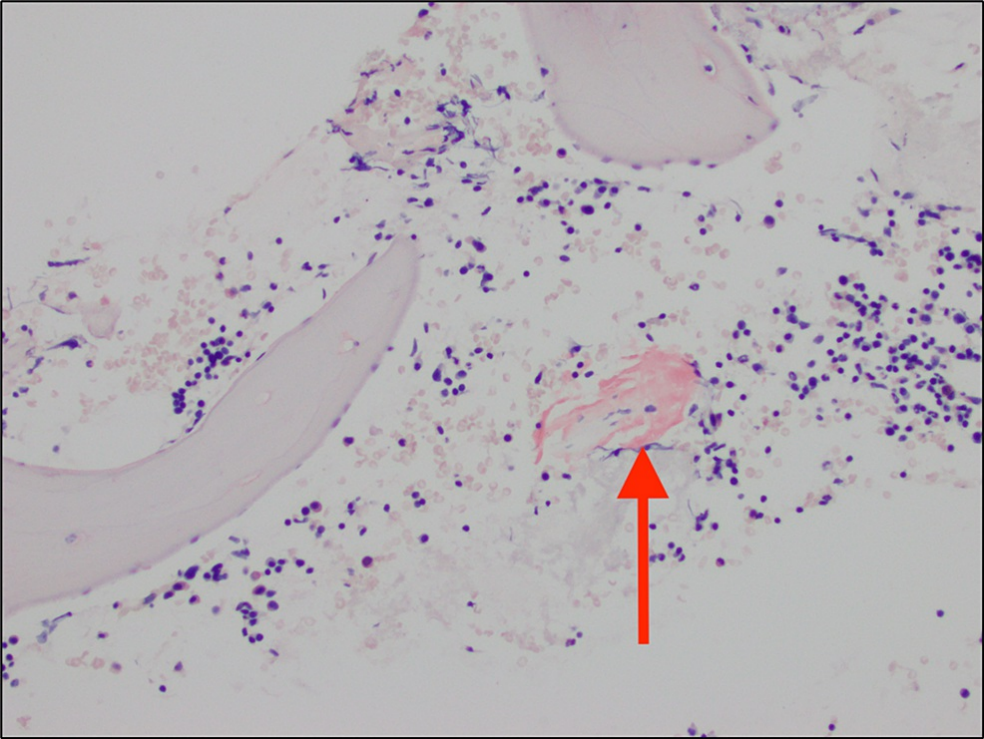
Bone Marrow Biopsy Congo-red stain is positive for amyloid deposit (shown in arrow).

A transthoracic echocardiogram was completed that demonstrated evidence of restrictive cardiomyopathy, including bi-atrial enlargement, dilated inferior vena cava, presence of increased right atrial pressure, and pulmonary hypertension with severe tricuspid insufficiency, all consistent with cardiac amyloidosis (Figure 3). The ejection fraction was 55-60% and the patient’s diastolic function could not be addressed due to atrial fibrillation. Right ventricular systolic function was mildly reduced and right ventricular systolic pressure was increased at 40-45 mmHg. Global longitudinal strain (GLS) was shown to be severely diminished. To understand the extent of infiltration within her cardiac tissue, magnetic resonance imaging (MRI) of the heart was completed (Figure 4). The cardiac MRI demonstrated subendocardial enhancement at the mid-ventricular and apical segment of the lateral and inferolateral wall with areas of enhancement within the septum, supporting cardiac involvement of amyloidosis. A bone marrow biopsy revealed amyloid infiltration and atypical monoclonal B cell lymphocytosis (Figure 5). These findings showcased that the patient had multisystemic AL amyloidosis that affected her heart and gastrointestinal system. A liver biopsy to assess for liver involvement with amyloidosis was offered, however, the patient denied this exam. The patient was informed of the treatment for AL amyloidosis and she expressed her wishes to be discharged home with at-home hospice care and further workup was ceased. In the following days, the patient went to an oncology clinic and was started on cyclophosphamide, bortezomib, and dexamethasone with daratumumab (CyBorD DARA) chemotherapy. A few days after starting chemotherapy, the patient was readmitted to the hospital for sepsis and was placed in the critical care unit, and expired.

## Discussion

Cardiac amyloidosis presents with diastolic dysfunction, decreased end ventricular volume, and restrictive cardiomyopathy [1]. Cardiac amyloidosis has a normal systolic ejection fraction until further progression of cardiac involvement [1]. The use of multi-imaging modalities is ideal, as a combination of a variety of features on imaging could all imply the diagnosis of amyloidosis [6]. The choice of imaging is usually echocardiography; however, cardiac MRI is used to determine infiltration into the ventricular wall [1,4]. Amyloidosis presents as a diffuse subendocardial enhancement on cardiac MRI [7,8]. Cardiac MRI is more sensitive and specific for cardiac ejection fraction and identification of cardiac amyloidosis before the presence of left ventricular hypertrophy [9]. However, neither cardiac MRI nor echocardiogram is able to differentiate between the different types of amyloidosis [4]. On further cardiac evaluation, approximately 50% of patients that have cardiac amyloidosis have low QRS voltage on ECG [7,10].

Laboratory values can assist in identifying early AL amyloidosis. Around 50% of patients with AL amyloidosis can present with abnormal urine or serum kappa to lambda ratio, an elevation of serum free light chain, or abnormal plasma cell differentiation [11]. Immunoglobulin cardiac toxicity results in the production, release, and elevation of B-type natriuretic peptides (BNP) [10]. Elevated cardiac troponin can also be seen in patients with amyloid cardiac involvement [11]. An elevation of troponin can be the result of myocardial ischemia from direct toxicity of light chain invasion on cardiac myocytes [12]. Damage to the cardiac myocytes can explain ECG changes, such as arrhythmia and conduction abnormalities [11]. Approximately 10-15% of patients with cardiac AL amyloidosis have atrial fibrillation [13]. Correction of arrhythmias by ablation has proved limited, with 83% of patients reverting to their arrhythmia shortly after correction within one year [13]. Anticoagulation is essential in avoiding thromboembolic events, such as strokes and myocardial infarction, in patients with persistent atrial fibrillation [12].

The conclusive treatment for AL amyloidosis includes the use of chemotherapeutic agents aimed at decreasing the monoclonal plasma cell proliferation to decrease the production of amyloid protein formation with the goal of delaying disease progression [13]. There are many chemotherapeutic agents available, however, IV melphalan has a 40% complete hematological response in patients one year after chemotherapy [13]. Many of these chemotherapy agents are associated with systemic toxicity, and therefore, poorly tolerated in patients with cardiac involvement [14]. These patients with advanced cardiac disease have a peri-treatment mortality risk of 30% if treated with chemotherapy, stressing the need for early AL amyloidosis detection [13]. Stem cell transplantation has shown a four-year survival rate of 90% for the treatment of AL amyloidosis [15]. For patients with cardiac involvement, successful stem cell transplantation has shown a median overall survival rate of more than 10 years [15]. Cardiac transplantation may be evaluated in specific cases; however, patients with AL amyloidosis may have greater mortality than patients of other disorders due to multisystemic disease progression, including the heart [13]. Therefore, AL amyloidosis is seen as a contraindication to transplant surgery by many transplant centers. With new and evolving therapies, early diagnosis of AL amyloidosis before advanced cardiac involvement is crucial in delaying disease progression and ensuring an early and appropriate response to treatment [13].

## Conclusions

AL amyloidosis is a rare disease with a nonspecific clinical presentation that proves difficult in diagnosing. The majority of patients that are diagnosed with AL amyloidosis have severe multisystemic disease in which treatment is less effective. “Red flag” symptoms, such as refractory atrial fibrillation, should raise suspicion for further evaluation of potential AL amyloidosis, as later physical manifestations of the disease can lead to delayed treatment with negative effects on patient outcomes. The treatment options for AL amyloidosis consist of chemotherapy, however, due to the vague clinical presentation of AL amyloidosis, treatment can often be delayed leading to poor patient outcomes. Therefore, with the emergence and improvement of medical therapy for AL amyloidosis, early diagnosis is critical for the potential of positive patient outcomes.
